# Potentially Inappropriate Prescribing and Potential Clinically Significant Drug–Drug Interactions in Older Outpatients: Is There Any Association?

**DOI:** 10.3390/medicina55070332

**Published:** 2019-07-03

**Authors:** Zorica Cvetković, Aneta Perić, Silva Dobrić

**Affiliations:** 1 Military Pharmacy “Slavija”, Central Pharmacy-Storage, Department for Military Health, 11000 Belgrade, Serbia; 2 Department of Pharmacy, Military Medical Academy, 11000 Belgrade, Serbia; 3 Medical Faculty of the Military Medical Academy, University of Defense, 11000 Belgrade, Serbia

**Keywords:** drug–drug interactions, older patients, outpatients, potentially inappropriate prescribing, STOPP criteria

## Abstract

*Background and Objectives:* The purpose of the study was to determine the prevalence rate of potentially inappropriate prescribing (PIP), by using the Screening Tool of Older Person’s potentially inappropriate Prescriptions (STOPP) criteria in older outpatients, and its association with potential clinically significant drug–drug interactions (csDDIs). *Materials and Methods:* A cross-sectional study included 248 outpatients ≥65 years old divided into two groups depending on the presence of csDDIs. For estimating the clinical significance of csDDIs we used Medscape′s "Drug Interaction Checker". We applied the thirty PIP indicators from the STOPP criteria. *Results:* The presence of PIP (25.00%; all patients) was significantly higher in the group with potential csDDIs compared to the other group (43 vs. 19, respectively; Chi-square test, χ^2^ = 9.947; *p* < 0.01). The most common PIP included the inappropriate use of proton pump inhibitors, long acting benzodiazepines, usage of thiazide diuretic in patients with gout, and duplication of therapeutic class. Patients with potential csDDIs had 43 potentially inappropriate medications (PIMs) prescribed. Out of this number, 12 (27.91%) PIMs were identified to participate in potential csDDIs. There was a correlation between the number of medications prescribed and the number of PIMs (ρ = 0.297; *p* < 0.01) and between the number of PIPs and the number of potential csDDIs (ρ = 0.170; *p* < 0.01). *Conclusions:* Older outpatients with potential csDDIs in relation to those with no potential csDDIs had significantly more prescribed drugs in total as well as inappropriate drugs. Almost 30% of these PIMs were included in potential csDDIs.

## 1. Introduction

The emergence of adverse drug reactions (ADRs) appears to be a serious and growing public health problem, which seems to be a lot more present in older people compared to the younger population [1]. Multimorbidity and polypharmacy are common in the elderly, so prescribing drugs for this group of patients presents a major challenge [2]. Namely, chronic diseases, physiologic changes associated with aging, and altered drug pharmacodynamics and pharmacokinetics as consequences of aging place elderly patients at a high risk of being prescribed potentially inappropriate medication (PIMs) [3,4,5,6]. On the other hand, the occurrence of ADRs is in a positive correlation with the number of concomitant medications (ranging from 10%, in the case of concomitant administration of two drugs, to 88% in the case of the administration of eight or more drugs), which makes interactions among them one of the main generators of ADRs, including very serious ones requiring hospitalization [7,8]. According to many international studies, the hospitalization rate due to ADRs, including those which are a consequence of drug–drug interactions (DDIs), varies between 2.4% and 16.6% among the elderly [9,10].

There are several methods for evaluating inadequacies in the prescription of medication to older patients. The Screening Tool of Older Person’s potentially inappropriate Prescriptions (STOPP) criteria refer to drugs classified according to the systems of the organs with which they operate [11,12,13]. The STOPP criteria contain a list of 65 drugs, which could exacerbate an existing condition in the elderly [11,12]. Potentially inappropriate prescribing (PIP) according to STOPP criteria is identified in 21–37% of older patients [14,15,16,17,18].

There are scarce data on the connection between PIP and potential clinically significant DDIs (csDDIs) in the elderly, although changes in the organism of the elderly as a consequence of the aging process place older patients at high risk for both csDDIs and PIPs. Due to this, the aim of our study was to determine the prevalence rates of PIP by STOPP criteria, as well as its connection with potential csDDIs in older outpatients. 

## 2. Materials and Methods

We conducted a cross-sectional study which included elderly patients (≥65 years), treated at the Military Medical Center (MMD) in Belgrade, who had two or more medications prescribed. Data collection took place during a period of two months. We looked over the medical documentations (drug prescriptions, medical records, and emergency medical records) of the 250 selected patients retrospectively, for six months, to determine chronicity of the drug use and processed the following data: Gender, age, number of prescribed drugs, types of drugs that were used, and indications for which a medicine was intended. For the classification of diseases, we used the "International Classification of Diseases—10th Revision" (ICD-10), and, for the classification of drugs, the Classification of Drugs, Anatomic–Therapeutic–Chemical Drug (ATC) Classification (20th ed., 2017) formulated by the World Health Organization Collaborating Centre for Drug Statistics Methodology [19]. The study did not include patients who had an acute illness in the past three months, patients with dementia and mental illness, as well as the patients who had cancer. 

In order to classify patients in the study and form cohorts, the drugs of all potential patients were checked for the possibility of clinically significant interactions with Medscape′s “Drug Interaction Checker” (http://reference.medscape.com/drug-interactionchecker). Interactions are divided into three groups: (A) Serious (requiring a change in the drug; the risk of possible ADRs overcomes the benefit of such therapy); (B) significant (requiring dose correction and careful monitoring of the patient); and (C) minor (not relevant). Category A and B interactions are considered clinically significant and were taken into further consideration. 

Based on the possible presence of csDDIs, patients were divided into two groups: Group I patients with at least one clinically significant interaction (category A and/or B interactions) and group II patients without the presence of any clinically significant interaction (without known interactions and/or category C interactions).

Afterward, all patients admitted to the study were tested for the adequacy of prescribing based on thirty PIP indicators (STOPP) and to determine the presence of PIP. The inadequacy of prescribed drugs was assessed on the basis of the above-stated STOPP criteria, using a shortened version that includes primarily drugs used in outpatient practice [20].

In statistical data processing, we used the software PASW Statistics (PASW Inc., Chicago, IL, USA) version 22 and Microsoft Excel^®^ 2010 (Microsoft Corporation, Redmond, WA, USA). The regularity of the distribution of continuous variables was checked using the Kolmogorov–Smirnov test. It turned out the parameters were not in normal distribution; therefore, we used Chi-square test to compare the rates of PIPs and potential DDIs. The significance of the correlation between the number of prescribed medications and csDDIs as well as PIMs, and PIMs and potential csDDIs was determined by one-tailed bivariate correlation (using Spearman’s ρ correlation coefficient). Statistically significant differences were considered at *p* < 0.05.

Members of the Ethics Committee of the Military Medical Academy (MMA) appointed according to the Decision of the Collegium of MMA Heads of Departments (Act of the MMA Deputy Head, con. no. 4494-1 dated 1 April 2016) approved the study on 1 February 2017). Informed consent was obtained from all individual participants included in the study.

## 3. Results

A detailed check of medical records revealed that two out of 250 patients had some of the factors that called for their exclusion from the study (one patient with dementia and one with cancer), which led to the drop of the final number of patients whose documentation was used in the study down to 248 (group I of 127 patients with potential csDDIs and group II of 121 patients without potential DDIs). The demographic and clinical characteristics of the patients participating in the study are given in Table 1.

There were no differences between these two groups of patients regarding age and gender. In both groups of patients, the age category 65–74 years was most prevalent. Additionally, in both groups, hypertension was the most prevalent diagnosis. Angina pectoris, diabetes mellitus type 2, prostate hyperplasia, and hypercholesterolemia were often present in both groups as well.

In group I, 638 potential csDDIs were identified (average number of csDDIs per patient was 5.02 ± 3.79, range 1–19). Patients in group I, had significantly more concomitantly prescribed drugs compared to those in group II (without potential DDIs) (total number: 880, average number per patient: 6.93; range: 2–15, median: 7 (interquartile range (IQR): 5–9) vs. total number: 423, average number per patient: 3.50; range: 2–7; median 3 (IQR: 3–4), respectively; *p* < 0.05) (Table 2). 

In both groups, those in the age category ≥85 years had a higher number of concomitantly prescribed drugs compared with those in the other two age categories (65–74 years and 75–84 years), but without statistically significant differences among them (Kruskal–Wallis test within groups: Group I χ^2^ = 1.971, *p* = 0.373; group II χ2 = 1.610, *p* = 0.477; all patients χ^2^ = 0.533, *p* = 0.766). However, the mean of prescribed drugs per patient was statistically higher in all age categories of patients in group I compared with patients in group II (Mann–Whitney U test of particular age category between groups: 65–74 years z = 8.467, *p* < 0.001; 75–84 years z = 5.731, *p* < 0.001; ≥85 years z = 2.866, *p* < 0.01) (Table 2).

By using thirty PIP indicators (STOPP criteria) we identified a total of 62 PIMs prescribed for 53 (21.37%) patients (Table 2). Forty-eight (20.97%) patients had one PIM and only five (2.02%) patients had two PIMs prescribed. In group I, a total of 43 PIMs was prescribed for 35 (28.35%) patients. Among them, 31 (24.41%) patients had one PIM and only four (3.15%) patients had two PIMs prescribed. In group II, a total of 19 PIMs was prescribed for 18 (14.87%) patients, out of which 17 (14.05%) patients had one PIM and only one (0.83%) patient had two PIMs prescribed (Table 2). It is obvious that patients with potential csDDIs had twice the number of PIMs prescribed than those without DDIs. This difference was significant *p* < 0.01 (Chi-square test, χ^2^ = 9.947).

Out of the 30 STOPP criteria that we considered, only four (13.33%) were shown to be relevant for identifying PIMs. They are given in Table 3. The higher prevalence of PIMs in the total number of patients who participated in the study related to proton pump inhibitors (PPIs) (39 of a total of 62 PIMs or 62.90%). In group I (patients with potential csDDIs), PPIs were inappropriately prescribed to 23 patients and in group II (patients without DDIs) to 16 patients. There were five PIMs related to the cardiovascular system and they were all prescribed to patients in group I. Patients in group I also had duplicate drugs prescribed to them. Out of a total of 62 PIMs, 10 were intended for the treatment of central nervous system disorders. Among those, seven were prescribed to patients in group I and three of them to the patients in group II (Table 3).

Based on the ATC classification of drugs, the 62 PIMs prescribed belong to one of the following groups: Primarily proton pump inhibitors (PPIs)—A02, medications that affect the renin–angiotensin system—C09, diuretics—C03, calcium channel blockers—C08, benzodiazepines—N05BA, and other antidepressants—N06AX. Regarding individual PIMs, we saw that pantoprazole (45.16%) was the most prescribed, followed by diazepam (16.13%) and omeprazole (12.90%), while other PIMs were less present (Table 4).

Analysis of 43 drugs that were potentially inappropriately prescribed according to STOPP criteria in group I showed that 12 (27.91%) of them participated in potential csDDIs. These 12 PIMs were involved in a total of 35 out of 191 (18.32%) potential csDDIs identified in patients of group I to whom PIMs were prescribed. Out of these csDDIs, six were serious and 29 were significant (Table 5). Three out of the eight PIPs with omeprazole were identified in five potential csDDIs (two serious and three significant interactions). Out of 28 inappropriate prescriptions with pantoprazole, only one was involved in a potential csDDI. On the other hand, valsartan as a PIM was prescribed only once, but it was identified in seven potential csDDIs (Table 5).

Pairs of drugs that give these 35 interactions are listed in Table 6. The most common combinations that led to csDDIs were: Omeprazole–digoxin (two serious and two significant interactions), bisoprolol–hydrochlorothiazide (two significant interactions), aspirin–hydrochlorothiazide (two significant interactions), bisoprolol–valsartan (two significant interactions), and hydrochlorothiazide–vitamin D (two significant interactions).

Correlation between the number of medications prescribed and the number of PIMs was significant (ρ = 0.297; *p* < 0.01). Furthermore, there was positive correlation between the potential csDDIs and the total of inappropriately prescribed drugs (ρ = 0.170; *p* < 0.01), as well as between the potential csDDIs and the total number of PIMs related to the central nervous system and those related to duplicate classes of medication (ρ = 0.198 and ρ = 0.185, respectively; *p* < 0.01).

## 4. Discussion

Polymorbidity and, consequently, polypharmacy characterized for older patients is recognized as the main generator of PIPs and potential csDDIs [21,22,23]. Our results confirmed these findings. In this study, patients with csDDIs had significantly more prescribed drugs than patients with no csDDIs (mean: 6.93, range: 2–15 vs. mean: 3.50; range: 2–7, respectively; *p* < 0.05), and there was a positive, significant correlation between the number of prescribed medications and the number of PIMs.

The average number of prescribed medications was 6.93 in the group of patients with potential csDDIs. For 26.77% of patients, there were more than nine medications in therapy, which is slightly more than in patients in the study by Fialova et al. [24].

The prevalence of PIMs in our study (21.37%) was higher than the overall prevalence in the “The Irish Longitudinal Study on Ageing” (TILDA) study (14.6%) [25], and similar to that found among older primary care patients in Serbia (27.3%) [26]. In contrast to both of these studies, where inadequate prescribing of proton pump inhibitors (PPIs) was not established, our results showed the highest prevalence of PPIs among PIMs (62.90%). This result is in line with the results reported by Forgacs et al., who showed that 25–70% of patients use these drugs contrary to the recommendations accepted worldwide [27]. The use of PPIs for a longer time interval in elderly people is associated with an increased risk of hip fracture, hospital infection with *Clostridium difficile*, and accelerated osteoporosis [14], and, in the future, there should be careful monitoring of older patients using PPIs in full doses longer than eight weeks for these adverse effects. Moreover, physicians must be informed on such outcomes with suggestion to stop long-term treatment with PPIs in these patients.

Although many studies have shown the association between the use of long acting (LA) benzodiazepines with a higher risk of falls and fractures in older patients, which has been emphasized for nearly three decades, almost all studies have shown that they continue to be used in older people worldwide [15,20,28,29]. Compared to the results from those studies, the use of LA benzodiazepines was 10% lower than in a study conducted in Serbia [21], almost three times lower than that reported by Bradly et al. [20], and similar to results obtained in a study conducted in Croatia [29]. Regardless of these differences in prescribing LA benzodiazepines to older people, these drugs are potentially inappropriate for them, and physicians have to be very careful when considering their use for this category of patients.

When looking at the results of some studies [25,30], we see that the prevalence of PIP in Serbia is somewhat higher. The reason for that might be a consequence of different STOPP criteria that were used in those studies in relation to the criteria used in our study. Such results support the opinion that each country should compile its list of STOPP criteria because there are significant differences in the national drug policies, habits in prescribing, and the availability of certain drugs [20,29,31].

This study indicated a presence of positive and significant correlation between the total number of inappropriate prescriptions among primary care older outpatients and the number of potential csDDIs as well as possible association between them. The associations between STOPP PIMs and preventable adverse drug reactions (ADRs) have recently been shown [20,31,32]. Fernández-Reguerio et al. reported that 69% of ADRs are related to PIMs according to STOPP or Beer’s criteria using the Naranjo Algorithm [33].

Our patients with potential csDDIs had 43 PIMs prescribed. Out of this number, 12 (27.91%) PIMs were identified to participate in potential csDDIs. Our study identified six and 29 drug–drug interacting pairs containing PIMs which potentially could give serious DDIs and significant DDIs, respectively. Among potential serious DDIs, the following drug–drug interacting pairs were identified: Omeprazole–digoxin, aspirin–ramipril, valsartan–lisinopril, aspirin–fosinopril, and valsartan–perindopril. Pharmacodynamics interactions were more frequently observed as compared to pharmacokinetic interactions. These findings are similar to other studies [34,35]. Pharmacodynamics interactions are observed when two drugs modify the effect of each other directly. A pharmacodynamics synergistic interaction may be desired by the prescriber if the two drugs potentiate the effect of each other as seen with valsartan–lisinopril, valsartan–perindopril, valsartan–bisoprolol, and perindopril–amiloride. A combination of an angiotensin II receptor antagonist and a beta blocker is clinically useful in a number of cardiovascular disorders. A combination of an angiotensin-converting enzyme (ACE) inhibitor and an angiotensin II receptor antagonist is recommended in treating patients with essential hypertension and left ventricular hypertrophy. Dual blockade of the renin–angiotensin system may increase risks of hypotension, hyperkalemia, and renal impairment. A study by McAlister et al. evaluated the combination of an ACE inhibitor and an angiotensin II receptor antagonist in elderly patients [36]. Results showed that for most of their elderly patients the benefits of combination therapy did not outweigh the risks which was confirmed by other authors, too [37]. These findings suggest that if patients do not have a recognized indication, a combination of an ACE inhibitor and an angiotensin II receptor antagonist, especially a combination of valsartan–lisinopril and/or valsartan–perindopril, should be avoided. Perindopril in combination with a potassium-sparing diuretic, like amiloride, can result in clinically relevant or severe hyperkalemia. Pharmacodynamic antagonism between aspirin and ACE inhibitors (ramipril, lisinopril, and fosinopril) can occur. A combination of ACE inhibitors and aspirin is often administered to improve outcomes in patients with left ventricular dysfunction, but potential negative interactions have called this approach into question. Aspirin may affect fluid homeostasis by decreasing the synthesis of renal prostaglandins and attenuate the antihypertensive effects of ramipril. Aspirin increases serum potassium level, and serum potassium level is altered by almost all antihypertensive drugs including ACE inhibitors, angiotensin II receptor antagonists, beta blockers, and diuretics [35,38,39]. To avoid serious DDIs (aspirin–ramipril and/or aspirin–fosinopril) those ACE inhibitors should be substituted with zofenopril. Zofenopril is a lipophilic, sulfhydryl-group-containing ACE inhibitor. The presence of this group provides vascular protection and anti-atherogenic effects due to the ability to reduce oxidative stress and activate the nitric oxide pathway; therefore its role in peripheral vascular function is independent of ACE inhibition [40,41]. A combination of hydrochlorothiazide and vitamin D may enhance the hypercalcemic effect of vitamin D [38].

On the other hand, pharmacokinetic interaction is seen when one drug alters the effects of the other drug at the level of absorption, distribution, metabolism, or excretion of the drug. In this study, such an interaction was seen at the metabolic level with the pantoprazole–clopidogrel combination, which is a very common interaction [36]. In two cases, a potentially csDDI was possible between omeprazole and digoxin. Namely, the long-term use of omeprazole will increase the serum concentration of digoxin by increasing gastric pH. Since digoxin has a small therapeutic width, the change in concentration can lead to the appearance of adverse effects of the drug [42]. If possible, omeprazole should be substituted with histamin-2 antagonists (e.g., ranitidine) because no interaction of these drugs and digoxin has been found using the drug interaction checker.

There were some limitations for this study. The number of patients we processed was not enough for generalization of results obtained to the whole older population even in Serbia, and, in the future, a study on prescription practice in the elderly in Serbia should be conducted as a national survey. Besides, in this study, we used a shortened version of the STOPP criteria with 30 indicators. This would be more acceptable for assessing inadequate prescribing in patients in primary health care, which may be the cause of a different incidence of PIP in our study compared to some larger studies. 

However, despite these limitations, the main goal of the study was achieved. We confirmed an association between PIMs and potential csDDIs. We showed that almost one-third of PIMs can participate in potential csDDIs.

## 5. Conclusions

The prevalence of PIM among older outpatients in our study was 21.37% and among those with csDDIs 33.1%. Out of these PIMs, almost one-third were included in drug–drug interacting pairs. These results indicate that general practitioners need to use different tools such as STOPP criteria and electronic drug–drug interaction checkers to aid in decision-making when prescribing drugs to patients, especially older ones with multimorbidity and polypharmacy. Healthcare professionals should pay special attention to long-term use of PPIs, long-acting benzodiazepines, presence of therapeutic duplications, and use of thiazide diuretics in patients with gout. Finally, the use of STOPP criteria in healthcare systems that do not have electronic support for recognizing drug–drug interactions can reduce potential clinically significant interactions in elderly patients.

## Figures and Tables

**Table 1:** Demographic and clinical characteristics of patients.

Characteristics	Group I (n = 127)	Group II (n = 121)	All Patients (n = 248)
**Gender, n (%)**			
male	79 (62.2)	75 (62.0)	154 (62.1)
female	48 (37.8)	46 (38.0)	94 (37.9)
**Age (years), mean ± SD (range)**	74.04 ± 6.50 (65–90)	74.60 ± 7.58 (65–97)	74.23 ± 6.92 (65–97)
**Age category (years), n (%)**			
65–74	71 (55.9)	64 (52.9)	135 (54.4)
75–84	46 (36.2)	44 (36.4)	90 (36.3)
≥85	10 (7.9)	13 (10.7)	23 (9.3)
**ICD-10 diagnosis, n (%)**			
I10	122 (49.19)	96 (38.71)	218 (87.90)
I20	34 (13.71)	15 (6.05)	49 (19.76)
I21	7 (2.82)	6 (2.42)	13 (5.24)
I50	22 (8.87)	4 (1.61)	26 (10.48)
Z95	10 (4.03)	1 (0.40)	11 (4.43)
E10	14 (5.64)	1 (0.40)	15 (6.04)
E11	47 (18.95)	9 (3.63)	56 (22.58)
E74	16 (6.45)	11 (4.43)	27 (10.89)
J44/J45	12 (4.84)	5 (2.02)	17 (6.85)
N40	26 (10.48)	39 (15.72)	65 (26.21)
K26	12 (4.84)	15 (6.05)	27 (10.89)
F32/41/42/48	16 (6.45)	0 (0)	16 (6.45)

Group I—patients with potential clinically significant drug–drug interactions (DDIs); Group II—patients without DDIs; ICD-10—International Classification of Diseases, 10th Revision: I10—hypertension, I20—angina pectoris, I21—myocardial infarction, I50—heart failure, Z95—presence of implants and grafts on the heart and blood vessels, E10—diabetes mellitus type 1, E11—diabetes mellitus type 2, E78—hypercholesterolemia, J45/J44—lung disease, N40—prostate hyperplasia, K26—peptic ulcer disease, F32/41/42/48—nervous diseases.

**Table 2:** Prescribed drugs and potentially inappropriate medications (PIMs) in the elderly patients.

Data	Group I (n = 127)	Group II (n = 121)	All Patients (n = 248)
Total number of prescribed drugs	880	423	1303
Mean of prescribed drugs per patient (range)	6.93 ± 2.64 (2–15)	3.50 ± 1.27 (2–7)	5.25 ± 2.70 (2–15)
Median (IQR) of prescribed drugs per patient	7 (5–9)	3 (3–4)	5 (3–7)
Mean of prescribed drugs by age category			
65–74 years	7.21 ± 2.74	3.41 ± 1.16	5.40 ± 2.86
75–84 years	6.43 ± 2.37	3.52 ± 1.44	5.02 ± 2.45
≥85 years	7.30 ± 3.02	3.85 ± 1.14	5.35 ± 2.74
Total number of PIMs	43 *	19	62
Total number (%) of patients with PIMs	35 (28.35)	18 (14.87)	53 (21.37)
Number (%) of patients with one PIP	31 (24.41)	17 (14.05)	48 (20.97)
Number (%) of patients with two PIPs	4 (3.15)	1 (0.83)	5 (2.02)

Group I—patients with potential clinically significant drug–drug interactions (DDIs); Group II—patients without DDIs; IQR—interquartile range; PIP—potentially inappropriate prescription; * *p* < 0.01 (Chi-square test, χ^2^ = 9.947).

**Table 3:** Potentially inappropriate medications (PIMs) identified by STOPP criteria.

STOPP Criteria	Number (%) of PIMs		Total Number (%) of PIMs
	Group I	Group II	All Patients
Cardiovascular system			
Thiazide diuretic in patients with gout	5 (11.62)	0 (0)	5 (8.06)
Central nervous system			
LT/LA benzodiazepines and with LA metabolites	7 (16.28)	3 (15.79)	10 (16.13)
Gastrointestinal system			
PPIs for PUD at full therapeutic dosage for >8 weeks	23 (53.49)	16 (25.81)	39 (62.90)
Duplicate class			
Two concurrent antidepressants	1 (2.32)	0 (0)	1 (1.61)
Two concurrent ACE inhibitors	2 (4.65)	0 (0)	2 (3.23)
Two concurrent calcium channel blockers	1 (2.32)	0 (0)	1 (1.61)
**Total number (%) of PIMs**	**43 (69.35)**	**19 (30.65)**	**62 (100)**

Group I—patients with potential clinically significant drug–drug interactions (DDIs); Group II—patients without DDIs, STOPP—Screening Tool of Older Person’s potentially inappropriate Prescriptions, PIP—potentially inappropriate prescriptions, LA—long acting, LT—long term, PPIs—proton pump inhibitors, PUD—peptic ulcer disease, ACE—angiotensin-converting enzyme.

**Table 4:** Potentially inappropriate medications (PIMs) prescribed to elderly patients.

Individual PIM	ATC Code	Number (%) of Prescribed PIMs
Omeprazole	A02BC01	8 (12.90)
Pantoprazole	A02BC02	28 (45.16)
Esomeprazole	A02BC05	3 (4.84)
Hydrochlorothiazide	C03AA03	2 (3.22)
Amiloride/hydrochlorothiazide	C03EA01	1 (1.61)
Felodipine	C08CA02	1 (1.61)
Perindopril/amlodipine	C09BB04	1 (1.61)
Ramipril/hydrochlorothiazide	C09BA05	1 (1.61)
Fosinopril/hydrochlorothiazide	C09BA09	1(1.61)
Perindopril/amlodipine/indapamide	C09BX01	1(1.61)
Perindopril	C09AA04	1(1.61)
Lisinopril/hydrochlorothiazide	C09BA03	1 (1.61)
Valsartan	C09CA03	1 (1.61)
Diazepam	N05BA01	10 (16.13)
Fluoxetine	N06AB03	1(1.61)
Trazodone	N06AX05	1 (1.61)
**Total number (%) of PIMs**		**62 (100)**

ATC—anatomic–therapeutic–chemical drug classification.

**Table 5:** Potentially inappropriate medications (PIMs) simultaneously involved in potential clinically significant drug–drug interactions (csDDIs).

Individual PIM	No. of Individual PIMs which Simultaneously Participated in csDDIs	% of PIMs which Simultaneously Participated in csDDIs (n = 43)	csDDIs		
			Serious	Significant	Total
Omeprazole	3	6.98	2	3	5
Pantoprazole	1	2.32	0	1	1
Hydrochlorothiazide	2	4.65	0	5	5
Amiloride/Hydrochlorothiazide	1	2.32	0	3	3
Felodipine	1	2.32	0	3	3
Ramipril/Hydrochlorothiazide	1	2.32	1	3	4
Fosinopril/Hydrochlorothiazide	1	2.32	1	3	4
Perindopril/Amlodipine/Indapamide	1	2.32	1	2	3
Valsartan	1	2.32	1	6	7
**Total**	**12**	**27.91**	**6**	**29**	**35**

**Table 6:** Pairs of drugs containing PIMs involved in potential clinically significant interactions.

Drug–Drug Pair Involved in Serious Interactions	Number of Interactions	Mechanism of Interaction			
		Pharmacokinetics	Pharmacodynamics	Potassium Level	Unknown
Omeprazole–digoxin	2	↑ effect ↑ pH			
Aspirin–ramipril	1		antagonism		
Valsartan–lisinopril	1		synergism		
Aspirin–fosinopril	1		antagonism		
Valsartan–perindopril	1		synergism		
**Total**	**6**	**2**	**4**	**0**	**0**
**Drug–Drug Pairs Involved in Significant Interactions**					
Bisoprolol–hydrochlorothiazide	2			↑↓ K	
Hydrochlorothiazide–indapamide	1			↓↓ K	
Omeprazole–digoxin	2		↑ toxicity		
Nebivolol–amiloride	1			↑↑ K	
Nebivolol–hydrochlorothiazide	1			↑↓ K	
Perindopril–amiloride	1		synergism		
Ramipril–aspirin	1		↔ toxicity		
Aspirin–hydrochlorothiazide	2			↑↓ K	
Bisoprolol–valsartan	2		synergism		
Valsartan–bisoprolol	1			↑↑K	
Valsartan–hydrochlorothiazide	1			↑↓ K	
Valsartan–aspirin	1			↑↑ K	
Valsartan–aspirin	1				↑ toxicity
Aspirin–valsartan	1		antagonism		
Pantoprazole–clopidogrel	1	↓ effect CYP2C19			
Bisoprolol–felodipine	1		↑↑ ACB		
Doxazosin–felodipine	1		↑↑ ACB		
Amlodipine–felodipine	1		↑↑ ACB		
Omeprazole–budesonide	1	↓ effect ↑ pH			
Metoprolol–hydrochlorothiazide	1			↑↓ K	
Hydrochlorothiazide–vitamin D	2		↑ effect		
Hydrochlorothiazide–metoprolol	1		↔ toxicity		
Fosinopril–aspirin	1		↔ toxicity		
Bisoprolol–indapamide	1			↑↓ K	
**Total**	**29**	**2**	**14**	**12**	**1**

↑↓K: First drug in pair increases and second, decreases serum potassium; ↓↓ K: Both drugs decrease serum potassium; ↑↑ K: Both drugs increase serum potassium; ↑: Toxicity ↑ pH: First drug increases the level of effect of the other by increasing gastric pH; ↑↔ toxicity: Either increases toxicity of the other; ↑ toxicity: First drug increase toxicity of other; ↔ toxicity: Either increases toxicity of the other by other;↓ effect CYP2C19: First drug decreases the effect of the other by affecting hepatic enzyme CYP2C19; ↑↑ ACB: Both increase antihypertension channel blocking; ↓ effect ↑ pH: First drug decreases the effects of the other by increasing gastric pH;↑ effect: First dug increases the effect of the other.

## References

[1] O’Mahony D., O’Sullivan D., Byrne S., O’Connor M.N., Rayan C., Gallagher P. (2015). STOPP/START criteria for potentially inappropriate prescribing in older people: Version 2. Age Ageing.

[2] Gallagher P., Barry P., O’Mahony D. (2007). Inappropriate prescribing in the elderly. J. Clin. Pharm. Ther..

[3] Mangoni A.A., Jackson S.H.D. (2004). Age-related changes in pharmacokinetics and pharmacodynamics: Basic principles and practical applications. Br. J. Clin. Pharmacol..

[4] Gallagher P.F., O’Connor M.N., O’Mahony D. (2011). Prevention of potentially inappropriate prescribing for elderly patients: A randomized controlled trial using STOPP/START Criteria. Clin. Pharmacol. Ther..

[5] O’Connor M.N., Gallagher P., O’Mahony D. (2012). Inappropriate prescribing. Drugs & Aging.

[6] Onder G., Landi F., Fusco D., Corsonello A., Tosato M., Battaglia M., Mastropaolo S., Settanni S., Antocicco M., Lattanzio F. (2014). Recommendations to prescribe in complex older adults: Results of the crıteria to assess appropriate medication use among elderly complex patients (CRIME) project. Drugs Aging.

[7] Leone R., Magro L., Moretti U., Cutroneo P., Moschini M., Motola D., Tuccori M., Conforti A. (2010). Identifying adverse drug reactions associated with drug-drug interactions: Data mining of a spontaneous reporting database in Italy. Drug Saf..

[8] Dechanont S., Maphanta S., Butthum B., Kongkaew C. (2014). Hospital admission/visits associated with drug-drug interactions: A systematic review and meta-analysis. Pharmacoepidemiol. Drug Saf..

[9] Sönnichsen A., Trampisch U.S., Rieckert A., Piccoliori G., Vögele A., Flamm M., Johansson T., Esmail A., Reeves D., Löffler C. (2016). Polypharmacy in chronic diseases–Reduction of Inappropriate Medication and Adverse drug events in older populations by electronic Decision Support (PRIMA-eDS): study protocol for a randomized controlled trial. Trials.

[10] Bertoli R., Bissig M., Caronzolo D., Odorico M., Pons M., Bernasconi E. (2010). Assessment of potential drug-drug interactions at hospital discharge. Swiss Med. Wkly..

[11] Hill-Taylor B., Sketris I., Hayden J., Byrne S., O’Sullivan D., Christie R. (2013). Application of the STOPP/START criteria: a systematic review of the prevalence of potentially inappropriate prescribing in older adults, and evidence of clinical, humanistic and economic impact. J. Clin. Pharm. Ther..

[12] Gallagher P., Ryan C., Byrne S., Kennedy J., O’Mahony D. (2008). STOPP (Screening Tool of Older Person’s Prescriptions) and START (Screening Tool to Alert doctors to Right Treatment). Consensus validation. Int. J. Clin. Pharmacol. Ther..

[13] Hanlon J.T., Schmader K.E. (2013). The Medication Appropriateness Index at 20: Where it started, where it has been and where it may be going. Drugs Aging.

[14] Ryan C., O’Mahony D., Kennedy J., Weedle P., Byrne S. (2009). Potentially inappropriate prescribing in an Irish elderly population in primary care. Br. J. Clin. Pharmacol..

[15] Cahir C., Fahey T., Teeling M., Teljeur C., Feely J., Bennett K. (2010). Potentially inappropriate prescribing and cost outcomes for older people: a national population study. Br. J. Clin. Pharmacol..

[16] Bradley M.C., Motterlini N., Padmanabhan S., Cahir C., Williams T., Fahey T., Hughes C.M. (2014). Potentially inappropriate prescribing among older people in the United Kingdom. BMC Geriatr..

[17] Urfer M., Elzi L., Dell-Kuster S., Bassetti S. (2016). Intervention to improve appropriate prescribing and reduce polypharmacy in elderly patients admitted to an internal medicine unit. PLoS ONE.

[18] Bruin-Huisman L., Abu-Hanna A., Van Weert H.C., Beers E. (2017). Potentially inappropriate prescribing to older patients in primary care in the Netherlands: A retrospective longitudinal study. Age Ageing.

[19] WHO Collaborating Centre for Drug Statistics Methodology ACT⁄DDD Index. http://www.whocc.no/atcddd/.

[20] Bradley M.C., Fahey T., Cahir C., Bennett K., O’Reilly D., Parsons C., Huges C.M. (2012). Potentially inappropriate prescribing and cost outcomes for older people: A cross-sectional study using the Northern Ireland Enhanced prescribing database. Eur. J. Clin. Pharmacol..

[21] Jyrkka J., Vartiainen L., Hartikainen S., Sulkava R., Enlund H. (2006). Increasing use of medicines in elderly persons: A five-year follow-up of the Kuopio 75 + Study. Eur. J. Clin. Pharmacol..

[22] Hovstadius B., Hovstadius K., Åstrand B., Petersson G. (2010). Increasing polypharmacy - an individual-based study of the Swedish population 2005-2008. BMC Clin. Pharmacol..

[23] Nyborg G., Straand J., Brekke M. (2012). Inappropriate prescribing for the elderly–A modern epidemic?. Eur. J. Clin. Pharmacol..

[24] Fialová D., Topinkova E., Gambassi G., Finne-Soveri H., Jonsson P.V., Carpenter I., Schroll M., Onder G., Sørbye L.W., Wagner C. (2005). Potentially inappropriate medication use among elderly home care patients in Europe. JAMA.

[25] Galvin R., Moriarty F., Cousins G., Cahir C., Motterlini N., Bradley M., Hughes C.M., Bennett K., Smith S.M., Fahey T. (2014). Prevalence of potentially inappropriate prescribing and prescribing omissions in older Irish adults: findings from The Irish LongituDinal Study on Ageing study (TILDA). Eur. J. Clin. Pharmacol..

[26] Vezmar Kovačević S., Simišić M., Stojkov Rudinski S., Ćulafić M., Vučićević K., Prostran M., Miljković B. (2014). Potentially inappropriate prescribing in older primary care patients. PLoS ONE.

[27] Forgacs I., Loganayagam A. (2008). Over prescribing proton pump inhibitors. BMJ.

[28] Lönnbro J., Wallerstedt S.M. (2017). Clinical relevance of the STOPP/START criteria in hip fracture patients. Eur. J. Clin. Pharmacol..

[29] Popović B., Quadranti N.R., Matanović S.M., Lisica I.D., Ljubotina A., Duliba D.P., Vlahović-Palčevski V. (2014). Potentially inappropriate prescribing in elderly outpatients in Croatia. Eur. J. Clin. Pharmacol..

[30] Yayla M.E., Bilge U., Binen E., Keskin A., Erol E., Yayla M., Bilge U. (2013). Ur The use of START/STOPP criteria for elderly patients in primary care. Sci. World J..

[31] Castillo-Páramo A., Claveria A., González A.V., Gómez-Serranillos I.R., Fernández-Merino M.C., Figueiras A. (2014). Inappropriate prescribing according to the STOPP/START criteria in older people from a primary care setting. Eur. J. Gen. Pr..

[32] Hamilton H., Gallagher P., Rayan C., O’Mahony D. (2011). Potentially inappropriate medications defined by STOPP criteria and the risk of adverse drug events in older hospitalized patients. Arch. Inter. Med..

[33] Fernández-Reguerio R., Fonesca-Aizpuru E., López-Colina G., Álvarez-Uria A., Rodriguez-Ávila E., Moris-De-La-Tassa J. (2011). Inappropriate drug prescription and adverse drug effect in elderly patients. Rev. Clin. Esp..

[34] Patel P.S., Rana D.A., Suthar J.V., Malhotra S.D., Patel V.J. (2014). A study of potential adverse drug-drug interactions among prescribed drugs in medicine outpatient department of a tertiary care teaching hospital. J. Basic Clin. Pharm..

[35] Jain S., Jain P., Sharma K., Saraswat P. (2017). A Prospective analysis of drug interactions in patients of intensive cardiac care unit. J. Clin. Diagn. Res..

[36] McAlister F.A., Zhang J., Tonelli M., Klarenbach S., Manns B.J., Hemmelgarn B.R. (2011). on behalf of the Alberta Kidney Disease Network The safety of combining angiotensin-converting-enzyme inhibitors with angiotensin-receptor blockers in elderly patients: a population-based longitudinal analysis. CMAJ.

[37] Anand S., Tamura M.K. (2012). Combining angiotensin receptor blockers with ACE-Inhibitors in elderly patients. Am. J. Kidney Dis..

[38] Baxter K., Stockley I. (2012). Stockley’s Drug Interactions: A Source Book of Interactions, Their Mechanisms, Clinical Importance and Management.

[39] Kothari N., Ganguly B. (2014). Potential drug–drug interactions among medications prescribed to hypertensive patients. J. Clin. Diagn. Res..

[40] Bucci M., Vellecco V., Cantalupo A., Brancaleone V., Zhou Z., Evangelista S., Calderone V., Papapetropoulos A., Cirino G. (2014). Hydrogen sulfide accounts for the peripheral vascular effects of zofenopril independently of ACE inhibition. Cardiovasc. Res..

[41] Borghi C., Omboni S., Novo S., Vinereanu D., Ambrosio G., Ambrosioni E. (2018). Efficacy and Safety of Zofenopril versus Ramipril in the treatment myocardial infarction and hearth failure. A review of published and unpublished data of the randomized double-blind SMILE-4 study. Adv. Ther..

[42] Sánchez-Fidalgo S., Guzmán-Ramos M.I., Galván-Banqueri M., Bernabeu-Wittel M., Santos-Ramos B. (2017). Prevalence of drug interactions in elderly patients with multimorbidity in primary care. Int. J. Clin. Pharm..

